# Editorial: Inflammation and cardiovascular disease: Vascular responses, mechanisms and therapeutic implications

**DOI:** 10.3389/fphys.2022.995175

**Published:** 2022-08-24

**Authors:** Nuria Villalba, George C. Wellman

**Affiliations:** ^1^ Department of Molecular Pharmacology and Physiology, University of South Florida, Morsani College of Medicine, Tampa, FL, United States; ^2^ Department of Pharmacology, University of Vermont, Larner College of Medicine, Burlington, VT, United States

**Keywords:** inflammation, endothelium, sepis, cardiovascular disease, calcium signaling

This special Research Topic in *Frontiers in Physiology* collates six original research articles examining novel pathways linking inflammation to cardiovascular disease (CVD). Inflammation is an essential bodily process mediated by the immune system to defend against bacteria, viruses, and other foreign entities. Acute inflammatory responses are often lifesaving with short-term effects typically manifested as local redness, swelling, heat and pain (Hannoodee and Nasuruddin, 2022). However, persistent chronic inflammatory states have been linked to a host of pathologies including rheumatoid arthritis, asthma, cancer, and cardiovascular disease—the leading cause of death world-wide (Soysal et al., 2020).

The vascular endothelium, the innermost layer of blood vessels, is profoundly impacted by activated inflammatory cells and the ensuing release of cytokines and growth factors. The healthy endothelium plays a pivotal role in maintaining cardiovascular health by 1) modulating vascular smooth muscle contractility, arterial/arteriolar diameter, peripheral resistance and regional blood flow; 2) controlling vascular permeability, the extravasation of fluids, blood components and accumulation of atherogenic low-density lipoprotein (LDL) within the vascular wall; 3) regulating the coagulation processes and thrombi/emboli formation. Fundamental molecular changes in endothelial cells occur swiftly with the onset of the inflammatory process resulting in the production of a variety of chemokines, cytokines, growth factors, and expression of adhesion molecules (Hunt and Jurd, 1998). Further, circulating mediators triggered by a proinflammatory host response, activate vascular endothelial cells causing vessel wall inflammation and increased endothelial permeability, which can profoundly impact cardiovascular function. Endothelial dysfunction is a major contributor and a sensitive indicator for CVDs such as hypertension, atherosclerosis, and stroke (Vanhoutte et al., 2017).

Recent evidence indicates that the transcriptional co-activators Yes-associated protein (YAP) and transcriptional coactivator with PDZ-binding motif (TAZ) may contribute to inflammatory endothelial cell dysfunction during hypertension (Daoud et al., 2022). Here, Xu et al. expand upon this concept using a mouse model of angiotensin II (AngII)-induced hypertension. They report that AngII infusion decreased levels of phosphorylated YAP/TAZ and promoted endothelial dysfunction as evidenced by impaired endothelial-dependent vasodilation, macrophage infiltration, and increased cytokine production in atherosclerotic plaques. Importantly, the authors show that selective inhibition of the YAP/TAZ pathway with verteporfin improved endothelial function and reduces vascular inflammation.

Calcium (Ca^2+^) is a critical second messenger regulating a multitude of endothelial cell signaling pathways. Small perturbances in intracellular Ca^2+^ signaling events within endothelial cells can translate into large physiological changes compromising responses ranging from vasodilation to coagulation. Although endothelial cell Ca^2+^ has been widely studied in animal models and cell culture systems, little work has been done in the human vasculature. Taylor et al. used confocal imaging and custom algorithmic analysis in human arteries to determine the spatio-temporal Ca^2+^ signaling signatures along the intima of peripheral arteries isolated from patients with or without existing CVD. Their initial findings revealed a complex pattern of dynamic endothelial cell Ca^2+^ signals that was markedly diminished in tissue exhibiting peripheral CVD. This work opens the door for the systematic study of the impact of inflammation and CVD on intrinsic Ca^2+^ signals in native endothelial cells of humans.

Three articles within this collection focus on vascular smooth muscle (VSM) function. Healthy, contractile VSM are circumferentially orientated within the vascular wall and dictate blood vessel diameter *via* contraction and relaxation in response to neurogenic, circulating, and locally produced vasoactive stimuli. Inflammation can profoundly impact VSM function, including a phenotypic switch from the contractile state to a proliferative/migratory state. Jeon et al. demonstrate increased in VSMC migration in response to HMGB1, a well-characterized danger-associated molecular pattern (DAMP) implicated in vascular inflammation and atherosclerotic plaque formation. Further, the authors show activator protein 1 (AP1)-induced osteopontin expression plays a critical role in HMGB1 mediated vascular migration.

Wang et al. present novel information on the effect of perivascular adipose tissue (PVAT) on vascular tone with aging. In previous work in murine models, the authors found that PVAT had paracrine effects causing the opening of potassium (K^+^) channels in VSMCs. Here, using a multidisciplinary experimental approach, including RNA sequencing, they show an age-dependent loss of dilatory actions of PVAT in mouse mesenteric arteries and describe post-translational alterations in signaling pathways involving the activation of Kv7 channels. Importantly, they show that aged PVAT exhibited upregulation of inflammatory signaling pathways, which could uncover new targets to improve cardiovascular health during aging.

In a separate study, Wang et al. investigated mechanisms underlying the beneficial effect of stellate ganglion block (SGB) to improve vascular reactivity following hemorrhagic shock. The authors demonstrated that the SGB inhibited post-hemorrhagic shock mesenteric lymph-induced vascular hypo-reactivity by reducing excessive autophagy in VSMCs. These results enriched the knowledge of mechanisms involved in vascular reactivity after hemorrhagic shock and provide experimental basis for clinical application of SGB in treating this pathology.

Rounding out this compendium, Meng et al. present their findings on sepsis-induced cardiomyopathy. Sepsis, a dysregulated host response to infection, can lead to multiple organ failure, with cardiomyopathy as one of the most common and serious complication. The vascular responses to sepsis include vasodilatory shock and impairment of cell oxygen utilization. Additionally, redox signaling attributes to the pathophysiology of sepsis. Here, the authors show that prophylactic treatment with rutin, a plant glycoside exhibiting antioxidant and anti-inflammatory actions, reduced mortality in a murine sepsis model. Further, they show that rutin pretreatment reduced sepsis-induced cytokine release, mitochondrial injury and apoptosis. This work lays the foundation for additional study examining the efficacy of rutin in the treatment of sepsis.

In summary, as debate persists regarding the role inflammatory processes play in the etiology of CVD, several anti-inflammatory drugs have shown beneficial effects in reducing cardiovascular risk. However, despite scientific advances over the past decades, we have only begun to scratch the surface of the complex relationship between inflammation and various forms of CVD. Understanding signaling pathways driving inflammation-induced phenotypic changes that occur within the vasculature is of great interest. As early diagnosis and effective treatments of CVD remain elusive, identifying inflammatory factors targeting the cardiovascular system and preventing their synthesis and/or release may offer promising alternatives in preventing the establishment and/or progression of various CVDs.
